# Content-Based Image Retrieval Using Spatial Layout Information in Brain Tumor T1-Weighted Contrast-Enhanced MR Images

**DOI:** 10.1371/journal.pone.0102754

**Published:** 2014-07-16

**Authors:** Meiyan Huang, Wei Yang, Yao Wu, Jun Jiang, Yang Gao, Yang Chen, Qianjin Feng, Wufan Chen, Zhentai Lu

**Affiliations:** 1 School of Biomedical Engineering, Southern Medical University, Guangzhou, Guangdong, China; 2 Laboratory of Image Science and Technology, Southeast University, the Key Laboratory of Computer Network and Information Integration (Southeast University), Ministry of Education, Nanjing, China; Banner Alzheimer's Institute, United States of America

## Abstract

This study aims to develop content-based image retrieval (CBIR) system for the retrieval of T1-weighted contrast-enhanced MR (CE-MR) images of brain tumors. When a tumor region is fed to the CBIR system as a query, the system attempts to retrieve tumors of the same pathological category. The bag-of-visual-words (BoVW) model with partition learning is incorporated into the system to extract informative features for representing the image contents. Furthermore, a distance metric learning algorithm called the Rank Error-based Metric Learning (REML) is proposed to reduce the semantic gap between low-level visual features and high-level semantic concepts. The effectiveness of the proposed method is evaluated on a brain T1-weighted CE-MR dataset with three types of brain tumors (i.e., meningioma, glioma, and pituitary tumor). Using the BoVW model with partition learning, the mean average precision (mAP) of retrieval increases beyond 4.6% with the learned distance metrics compared with the spatial pyramid BoVW method. The distance metric learned by REML significantly outperforms three other existing distance metric learning methods in terms of mAP. The mAP of the CBIR system is as high as 91.8% using the proposed method, and the precision can reach 93.1% when the top 10 images are returned by the system. These preliminary results demonstrate that the proposed method is effective and feasible for the retrieval of brain tumors in T1-weighted CE-MR Images.

## Introduction

In the medical field, digital images are produced every day and are used by radiologists to make diagnoses. However, searching for images with the same anatomic regions or similar-appearing lesions according to their visual contents in a large image dataset is difficult. Content-based image retrieval (CBIR) is presented as a possible and promising solution to indexing images with minimal human intervention [1]. In the medical field, CBIR mainly serves for two types of application: retrieval of the same anatomic regions [2]–[4] and retrieval of clinically relevant lesions (e.g., lesions of the same pathological category) [5]–[8]. Category retrieval is only used when lesions of the same pathological category are expected to be retrieved.

The current study aims to develop a CBIR system for retrieving T1-weighted contrast-enhanced MR (CE-MR) images that contain brain tumors of the same pathological category. The goal of the proposed CBIR system is to aid diagnosis for the given query images. Concretely, the most similar tumors with the same pathological category in the dataset are returned when a tumor is sent to the system as a query. Users can then choose the most relevant images and access their associated diagnosis information to support the diagnosis for the current case. This study focuses on three types of brain tumors with high incidence rates in clinics: gliomas, meningiomas, and pituitary tumors, percentages of which are about 45%, 15%, and 15% of all brain tumors, respectively.

Several studies have developed CBIR systems for brain tumors in MR Images. Huang et al. [9] presented a diagnostic support tool for pediatric brain diseases using the segmented lesion as a query to retrieve the most similar lesions in the database. Dube et al. [10] proposed a methodology for the image retrieval of glioblastoma multiforme (GBM) and non-GBM tumors on MR Images. Moustakas et al. [11] presented a two-tier CBIR architecture for regions of interest (ROI) of brain MR Images; this architecture was evaluated against a dataset comprising T1, T2, and PD MR scans. As opposed to other MR modalities used in the previous studies, the category retrieval of brain tumors is performed on the T1-weighted CE-MR Images in this study.

**Figure 1:**
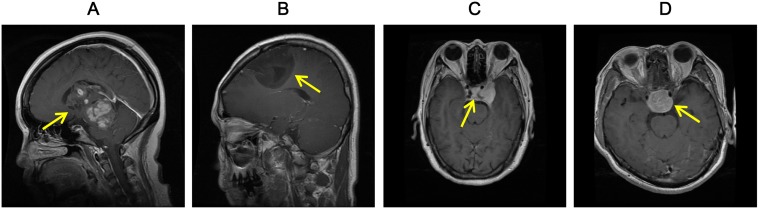
Four examples of brain tumors in T1-weighted CE-MR Images. The tumors are indicated by the yellow arrows in each image. (A) and (B) Gliomas in different subjects having dissimilar appearances. (C) A meningioma and (D) a pituitary tumor from different subjects showing a similar appearance.

In general, feature extraction and distance metrics in the feature space are two crucial factors for CBIR. First, different appearances of brain tumors in MR Images demand more discriminative features. For instance, tumors with the same pathological category but from different patients or at different disease stages exhibit different appearances on the images [Figures 1(A) and 1(B)]. Conversely, lesions of different pathological categories may show visual similarity [Figures 1(C) and 1(D)]. Given that low-level visual features, such as color (intensity), texture [12]–[17], and shape [18], [19], are not adequately discriminative to describe high-level semantic concepts [20], additional distinctive features are highly desirable [6], [7]. Second, learned distance metrics, which are capable of reducing the semantic gap between visual features and semantic concepts [21], have been investigated intensively and widely used for CBIR [6], [22], [23].

**Figure 2:**
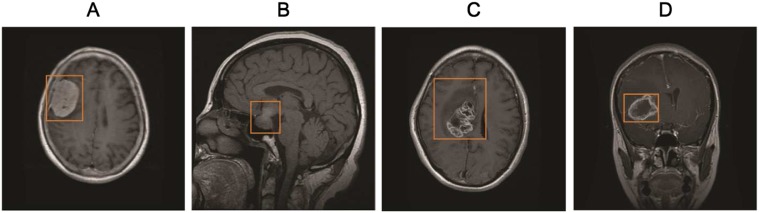
Illustrations of four typical brain tumors with rectangles as ROIs (orange lines) in T1-weighted CE-MR Images: (A) A meningioma located near the skull, (B) a pituitary tumor located near the sphenoidal sinus, (C) a glioma containing edema and necrosis, and (D) a glioma surrounded with edema.

Recent studies [5]–[7] have verified that image features constructed by bag-of-visual-words (BoVW) model are more discriminative than other commonly used intensity and texture features for medical images. We previously used a marginal information descriptor and region-specific BoVW to obtain structural information on tumors and their surrounding regions [6], [7]. Tumor regions were partitioned into several sub-regions, and the image contents in each sub-region were represented using the BoVW-based approach. However, the tumor regions were outlined manually in these studies, which is not a trivial task and could cause intra- and inter-operator variations [24]. To promote the feasibility of the proposed CBIR system, rectangular ROIs (Figure 2) are used in the current study instead of tumors contours for feature extraction; this method demands a more subtle extraction approach to integrate the spatial information into the BoVW-based image feature. Several other approaches have addressed this problem for object recognition tasks [25]–[28]. The spatial pyramid method [25], for example, is a representative of these methods. The spatial pyramid method symmetrically partitions the image into uniform cells at a series of resolution levels (i.e., 1×1, 2×2, and 4×4) and then combines all of the BoVW histograms in each cell to incorporate global and local information. With the help of spatial layout information to improve the discriminative power, the spatial pyramid method outperforms the basic BoVW model and has thus been employed in several studies [29], [30]. However, compared with the symmetrical partitioning in the spatial pyramid method, the partition can be learned to make the feature more discriminative [28] when the contents of the images from a specific category are distributed with intrinsic regularity. Brain tumor T1-weghted CE-MR Images show that the layouts of the tumor, edema, and surrounding normal tissue have strong correlations [6]. For instance, meningiomas and pituitary tumors are homogeneous lesions [Figures 2(A) and 2(B)]; these two tumor types are usually not associated with edema. Meningiomas are usually adjacent to the skull, gray matter, and cerebrospinal fluid. Pituitary tumors are adjacent to the sphenoidal sinus, internal carotid arteries, and optic chiasma. By contrast, gliomas are inhomogeneous lesions and usually surrounded by edema [Figures 2(C) and 2(D)]. Therefore, learning a spatial partition of the ROI in the current study is reasonable for obtaining discriminant features.

The contributions of the current study can be summarized as follows:

1. A partition learning algorithm is proposed based on the concept that the best partition will lead to the largest difference among the BoVW histograms of the sub-regions. This idea allows the region with variable appearance to be partitioned and image contents in each partitioned sub-region to be consistent; thus, combinational histograms of sub-regions can bring more discriminative information. We present a novel objective function of the partition learning method, provide an optimization approach, and evaluate the effectiveness of the method in the CBIR of brain tumor T1-weighted CE-MR Images.
2. A distance metric learning approach, called Rank Error-based Metric Learning (REML), is introduced to reduce the semantic gap between high-level semantic concepts and low-level visual features in the proposed CBIR system. A novel objective function that integrates rank error is proposed, and a stochastic gradient descent-based optimization strategy is presented to find the optimal solution of the objective function. REML can project image features to a low-dimensional feature space, where the learned distance is expected to reflect the differences between the semantic concepts.

## Materials and Methods

### 2.1 Ethics Statement

This study was approved by the Ethics Committees of Nanfang Hospital and General Hospital, Tianjin Medical University. Patient records/information was anonymized and de-identified prior to analysis.

### 2.2 Image Data

At present, only a certain number of slices of brain T1-weighted CE-MR with a large slice gap, not 3-D volumes, are usually acquired and available for clinical practice in China. Considering the difficulty to build a 3D model using such sparse data, we opted to build a 2D image-based CBIR system for clinical applications. In our implementation, we selected slices from 3D images primarily based on the visible size of tumors. The slice containing the largest tumor size and its adjacent 1 to 10 slices were selected as the typical slices for constructing our dataset. Slices in different views (transverse, coronal, and sagittal) were processed together in the proposed CBIR system. For instance, when one slice in a specific view was fed into the system as a query, slices containing tumors with the same category may be expected to be returned even when they are in different views. We expect that the retrieval method would only work in realistic situations when a certain number of 2D slices in different views were acquired for clinical practice.

In this study, the brain T1-weighted CE-MR dataset was acquired from Nanfang Hospital, Guangzhou, China, and General Hospital, Tianjin Medical University, China, from 2005 to 2010. This dataset consisted of 3064 slices from 233 patients. Each slice is 512×512 pixels, and the pixel size is 0.49 mm×0.49 mm. Three types of brain tumors, namely, meningiomas, gliomas, and pituitary tumors, are apparent in the dataset (Table 1). For each patient, three experienced radiologists initially consulted the patient pathology report to obtain the pathology type and then labeled the images. Each radiologist dealt with all images independently. Afterward, the radiologists discussed and reached a consensus regarding the label of every tumor in each image. In the current study, two images containing tumors of the same category were defined to be relevant (similar); otherwise, they were considered irrelevant (dissimilar).

**Table 1:** Summary of the image dataset.

Tumor category	Number of patients	Number of slices	View	Number of slices
Meningiomas	82	708	Transverse	209
			Sagittal	231
			Coronal	268
Gliomas	89	1426	Transverse	494
			Sagittal	495
			Coronal	437
Pituitary tumors	62	930	Transverse	291
			Sagittal	320
			Coronal	319

### 2.3 BoVW Model

The BoVW model has been widely used in image classification and CBIR [4], [31]–[33]. It involves four steps. First, patches represented by the local descriptors are sampled in each ROI in an image of the given image dataset. Second, a dictionary is learned by a clustering algorithm, and each of the cluster centers is a visual word. Third, local descriptors of a ROI in a new image are quantized to the learned dictionary by a coding method. Finally, a BoVW histogram is constructed to represent the ROI by a pooling method.

In the BoVW framework, selections of the patch sampling method and the local descriptor are two basic tasks. As introduced in our previous study [7], raw patches sampled in a dense fashion are applicable for capturing discriminative information in brain tumor T1-weghted CE-MR Images. Therefore, in this study, we retain the use of raw patches as the local descriptor and sample these patches in a dense manner. After the patch sampling step, a dictionary can be learned from the sampled patches by a clustering algorithm. In this study, we used the *k*-means clustering algorithm to learn *V* visual words in a dictionary *D* because of its simplicity and effectiveness.

For a ROI in a new image, the local descriptor of each pixel in this ROI can be mapped to *D*, and a code for a local descriptor is generated; this process is called coding. A basic coding method in the BoVW approach is done by assigning an image patch to one visual word, also called hard-assignment. In the hard-assignment method, a patch is mapped to a visual word that is most similar to the current patch. If hard-assignment is used for coding, each code has only one non-zero element. This coding method may ignore all ambiguity regarding the meaning of a patch. Unlike hard-assignment, soft-assignment assigns a degree of similarity to each of the visual words, and a small group of elements in a code can be non-zero. This coding method can help in modeling the inherent uncertainty of the image patch, while considering the continuous nature of the image patches [34]. Aside from soft-assignment, sparse coding, such as locality-constrained linear coding [35], is another alternative to hard-assignment.

To represent a ROI in an image, all coding results over *m* spatial regions of the ROI are aggregated, and an *mV*-dimensional vector 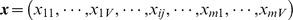 is obtained. This process is called pooling. Each dimension of the pooled feature 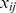 is achieved by taking the *j*th visual word of all codes 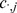 in the specific spatial region *R_i_*, and performing a predefined operator on the set of visual words. Therefore, 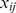 can be defined as follows:
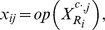
where 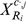 is the set of visual words of codes 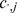 in the spatial region *R_i_*. Generally, 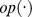 is the operator for computing the statistics of the visual words under the *p*-norm; thus, 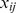 can be calculated by:



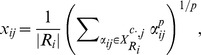
when *p* = 1, 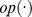 is the average pooling, and when 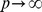, 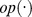 is the max pooling.

In the basic BoVW model, the whole ROI is considered as a spatial region, and all codes in the spatial region are pooled together; thus, the spatial information is ignored.

### 2.4 Proposed Method

**Figure 3:**
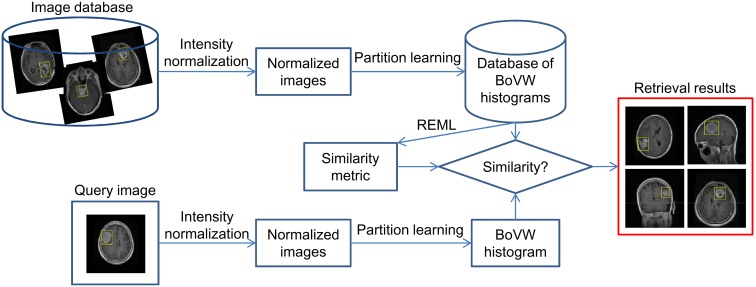
Flowchart of the proposed CBIR system.

The proposed CBIR system mainly contains two components. (1) Offline feature extraction and (2) online image retrieval. In (1), a bounding box is first set to cover the tumor region in each image in the database as the ROI. The proposed partition learning method is used to partition the ROI into a set of sub-regions according to the largest difference among the BoVW histograms of these sub-regions. Thus, the BoVW histograms that best capture the discriminative information can be built by concatenating the histograms in the partitioned sub-regions and saved into the database. According to the BoVW histograms extracted from the ROIs with known categories, an optimized similarity metric is learned for subsequent online image retrieval using the proposed REML method. In (2), a bounding box is also needed to cover the tumor region in a query image. The BoVW histogram is built online using the learned partitioned sub-regions and fed to the CBIR system. By calculating the similarity between the BoVW histograms of the query image and each image in the database according to the learned similarity metric, the CBIR system returns the images that are relatively similar to the query image. The flowchart of the proposed CBIR system is shown in Figure 3.

### 2.5 Intensity Normalization

In this study, we use the raw intensity as local patch descriptor to construct the BoVW histogram, which heavily relies on pixel intensities. However, image intensities in MR Images do not have a fixed meaning and vary considerably within or between subjects. Therefore, an intensity normalization step is needed to preprocess the MR Images. First, images were fed to an implementation of N3 bias field correction algorithm [36] to deal with the intensity heterogeneity. The intensities were then normalized as follows: intensity values at the 1% and 99% quantiles are computed for the brain region and then these two values are used to scale the intensities to [0, 1] through the min–max method.

### 2.6 Partition learning

As described in Section 1, while the spatial pyramid method outperforms the basic BoVW model, spatial partitions are fixed without considering the empirical or theoretical knowledge of the objects in the image. Therefore, a novel method is proposed to learn the optimal partitions of the spatial regions for pooling in this section.

**Figure 4:**
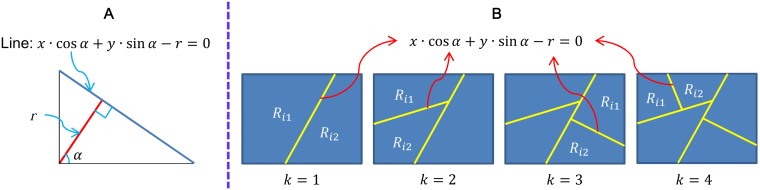
(A) Normal form of a linear equation. (B) Formation of the spatial partition patterns by successive partition of regions.

Given a ROI in a brain tumor T1-weghted CE-MR Image, a series of lines are used to separate this ROI to sub-regions [Figure 4(B)]. The learned partitions are decided by finding the largest difference among the BoVW histograms of the sub-regions. Based on this idea, we use the normal form of a linear equation to represent a line to partition an image [Figure 4(A)]. In addition, the Kullback–Leibler divergence is used to measure the difference between the BoVW histograms of two sub-regions; this divergence has been proven to be effective in discriminating two different classes [37]. Therefore, the objective function of the partition learning can be defined as follows:
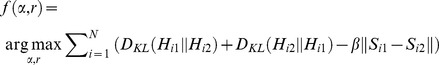
(1)where 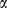 and *r* are two parameters in a linear equation [Figure 4(A)]; 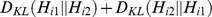 is the symmetrized Kullback–Leibler divergence; *N* is the image number in the dataset; 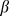 is a parameter that controls the trade-off between the maximized divergence values and the area constraint; 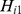 and 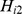 are the BoVW histograms in regions 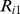 and 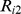, respectively; and 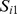 and 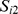 represent the areas of regions 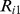 and 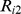, respectively. Moreover, the constraint of the areas of the two sub-regions in Eq. (1) is employed to ensure similar areas in the two sub-regions because the partition is meaningless when one partitioned area has a much smaller area than the other partitioned area. For an extreme example, when one partitioned area only contains one pixel and the other partitioned area contains significantly more pixels, the divergence values may be large; however, this partition cannot capture useful information from the image.

To optimize the objective function, we first define the ROIs of the images. For each brain tumor T1-weghted CE-MR Image, outlining the tumor by hand is not practical. Therefore, we locate a bounding box to cover the tumor region in each image. For a tumor region in an image, the bounding box is generated manually by specifying the opposite corner of a rectangle. The relative locations between the bounding box and the tumor region can be different for each image in our experiment. Starting with the bounding box as the spatial partition with one region, we partition the region further into two parts with a line. A disjoint partition set denoted as 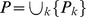 is obtained after *k* successive partitions. When the number of partitions is equal to *k*, the number of the separated regions is 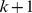. In particular, the bounding box is the case where 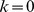. For each partition, we aim to find a line to separate a given region into two sub-regions [Figure 4 (B)] that can maximize the difference between these two sub-regions.

In the current study, the coordinate descent-like iterations and greedy forward selection methods are used to approximately optimize Eq. (1). The parameters 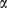 and *r* in a linear equation are treated as two sets of variables in which alternating coordinate descent-like iterations are performed to find the best partition for a given region. To increase the number of partitions, greedy forward selection is used to compute the next best partition by selecting the parameters that increase the objective function the most. The *k*th partition occurs in the region with the maximal area among the *k* regions. The complete partition learning procedure is listed in Table 2.

**Table 2:** Algorithm 1.

**Input:**
Bounding boxes of brain tumor T1-weghted CE-MR Images 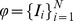 ; Maximum iteration number *t_max_*.
**Output:**
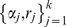 (Final solution of the partition learning method).
▪ Initialize 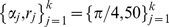 and the step sizes 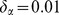 and 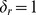 .
▪ For 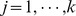
Find the region with maximal area among *k* regions.
** For** *t* = 1,…, *t_max_*
▪ Initialize the parameters:
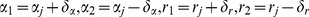
▪ Calculate 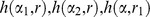 and 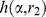 , respectively, using:
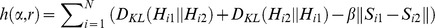
▪ Optimize Eq. (1) using:
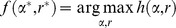
▪ Update 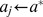 and 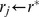

Compared with the spatial pyramid method that pre-sets a fixed partition for all bounding boxes, the proposed partition learning method generates a set of spatial partitions based on maximizing the difference between the two sub-regions. This idea is similar to coarse segmentation, which isolates the objects from their background according to their dissimilarities [38]. In addition, instead of using a pyramid, only the final partition set 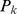 generated by *k* partitions is used as we expect the partition to adjust its resolution depending on the spatial distribution of discriminative information for the images. Although this idea is straightforward, it contributes to a considerable improvement in retrieval accuracy in the experiment. Moreover, the proposed partition learning method can use empirical or theoretical knowledge of the brain tumors in T1-weghted CE-MR Images to discover descriptive partitions and better present the spatial configuration of the semantic components of the brain tumors in T1-weghted CE-MR Images.

### 2.7 Distance Metric Learning

When a query image is presented, the similarity between the feature vectors (BoVW histograms in this study) of the query image and the images in the dataset is measured. Image representation by visual features usually results in information loss, that is, the performance of a CBIR system suffers from the gap between the low-level visual features and the semantic concepts. Furthermore, a CBIR system cannot perform well when common distance metrics, such as the Euclidean distance or χ^2^ distance, are used to measure the similarity of the features because BoVW histograms may not be located in the Euclidean space. Different visual words have different contributions to image similarity, whereas Euclidean distance provides the same weight to all visual words. Moreover, visual words in BoVW histograms may be relative. Therefore, a distance metric learning method is used to find a linear transformation that projects the image features into a new and meaningful feature space to reduce this semantic gap. Previous studies show that well-designed distance metrics can perform better than the Euclidean distance on the CBIR system [39], [40].

**Figure 5:**
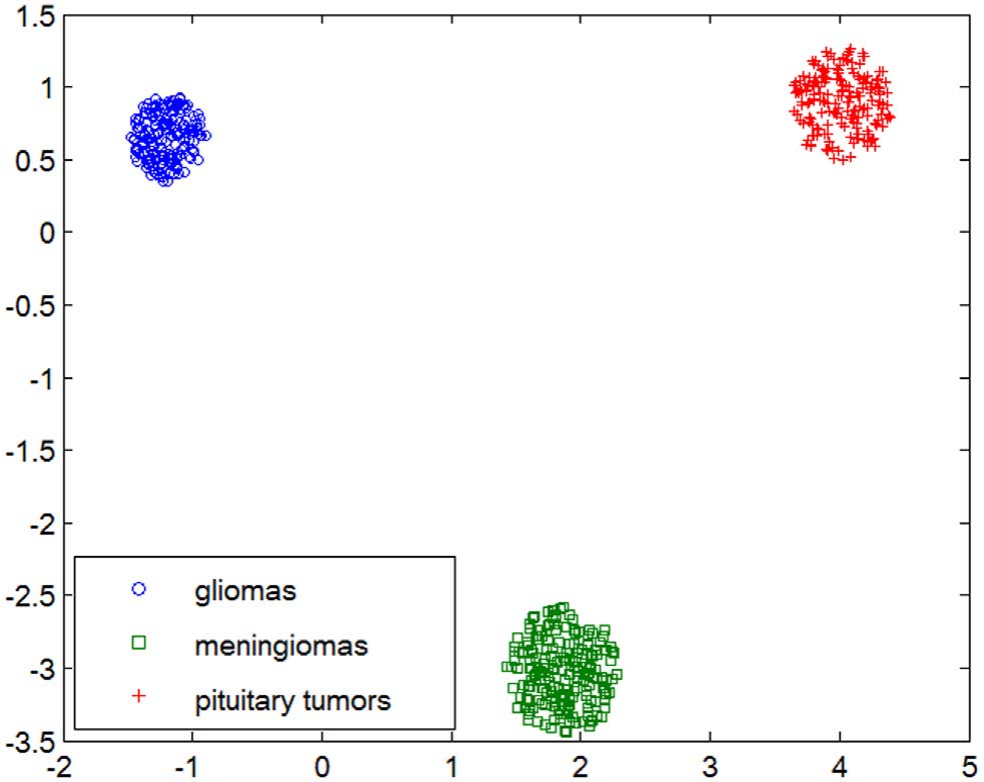
BoVW histograms of brain tumor T1-weghted CE-MR Images are projected into 2-dimentional feature space by REML. In the training dataset, three types of brain tumors are linearly separable in the transformed space learned by REML.

The squared Mahalanobis distance is used to compute the similarity between the feature vectors ***x*** *_i_* and ***x*** *_j_*:
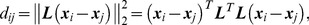
(2)where 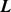 is a transformation matrix of size *d*×*D*, where *d* and *D* are the dimensionalities of the transformed and the original feature space, respectively. In brief, the distance metric learning problem can be formulated as
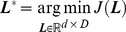
(3)where *J*(***L***) is an objective function. An optimal projection 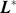 can be obtained to project the image features to a new feature space by minimizing this objective function, making the squared Mahalanobis distance between data with the same labels closer and that with different labels farther (Figure 5).

A number of distance metric learning algorithms have been proposed to find an optimal ***L*** for minimizing an objective function; examples of these algorithms include kernel-based distance metric learning [22], Xing’s method [41], local fisher discriminant analysis (LFDA) [42], large margin nearest neighbor (LMNN) [43], and close-form metric learning (CFML) [44]. Most of these algorithms are designed to achieve high classification accuracy. However, the evaluation measures for CBIR systems are very different from those for classification. In our previous study [6], a distance metric learning method called maximum mean average precision projection (MPP) was proposed to optimize retrieval measures directly. We also verified that the MPP method yields good performance compared with other approaches [6]. In this study, we propose a novel metric learning method based on the minimization of rank error to achieve the projection ***L***.

#### 2.7.1 Objective function

In this study, two images with tumors of the same category are considered relevant; otherwise, they are considered irrelevant. Given a query image *I_q_* with feature vector *x_q_*, all images in the dataset are retrieved and sorted in a ranking list according to increasing order of the squared Mahalanobis distance 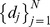, where *d_j_* is the distance between *x_q_* and *x_j_*, and *N* is the image number in the dataset. Meanwhile, 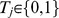 (1 for relevant and 0 otherwise) is used to represent the relevance between *I_q_* and an image *I_j_* in the dataset. For a set of retrieved images 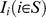 with 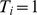 and a set of retrieved images 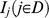 with 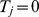, where *S* is the set of relevant pairs and *D* is the set of irrelevant pairs, *d_i_* is made smaller so that 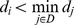. Therefore, the loss function can be defined based on the rank error of the relevance:
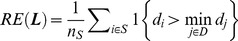
(4)where 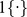 is the indicator function. The distance gap of ***x*** *_i_* is defined as



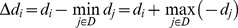
(5)Thus, Eq. (4) can be rewritten as
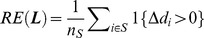
(6)


Given the max function in Eq. (5), the distance gap is noncontinuous and nondifferentiable. The max function can be approximated using the log-sum-exp method [45]:
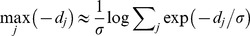
(7)where 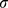 is the scaling parameter and set to 1 in this study. The indicator function in Eq. (6) is also noncontinuous and nondifferentiable; thus, the logistic loss function can be used to approximate the indicator function [46] and is defined as



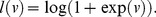
(8)The loss function can be approximated, and can become continuous and differentiable (denoted as 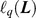):
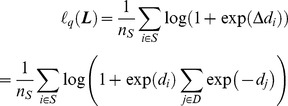
(9)


If 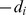 is represented as a ranking function, 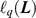 can be transformed to the loss function of “IR Push” [46]. To the best of our knowledge, the approximation of rank error has not been used in distance metric learning.

Based on the loss function, the proposed objective function is defined as follows:
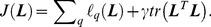
(10)


On the right hand side of Eq. (10), the second term is the regularization term, where 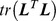 is the trace of matrix 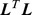. This term ensures the sparsity of the matrix 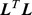 and makes the solution more reliable [47]. Parameter 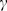 is used to control the trade-off between the empirical loss and the regularization term, which can be set empirically or estimated by cross-validation.

#### 2.7.2 Optimization method of the objective function

To achieve the optimized projection ***L***, the objective function defined in Eq. (10) should be minimized. The gradient-based optimization method is used to find the optimal solutions; thus the following issues should be considered.

(1) The computation of 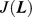 or its gradient over all query pairs is time-consuming and impractical on a large training dataset. Therefore, the stochastic gradient descent algorithm is applied in this study for optimizing 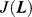; this algorithm exhibits reasonable performance in the experiments. The gradient of 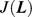 in Eq. (10) with respect to 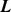 is
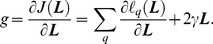
(11)


Define 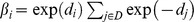, then Eq. (9) can be rewritten as
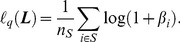
(12)


Now, the gradient of 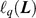 with respect to 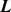 can be defined as
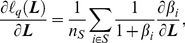
(13)where



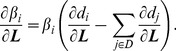
(14)Define 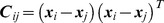, then Eq. (3) can be rewritten as
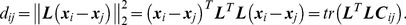
(15)


Therefore, the gradient of 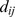 with respect to 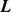 is
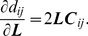
(16)


(2) Given the non-convexity of 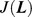, the final solution of ***L*** is highly dependent on the initial estimation ***L***_0_. The initial estimation ***L***_0_ is expected to be very close to the global optimal solution. Some other metric learning algorithms can be applied to obtain ***L***_0_. As reported in our previous study [6], LFDA [42] can be used to obtain an appropriate linear transformation with relatively high retrieval performance. Thus, LFDA is also used to estimate the initial ***L***_0_ in this study. The LFDA projection matrix ***L***_LFDA_ can be defined as:
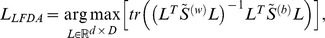
where



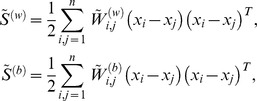
and *tr*() denotes matrix trace. 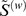 and 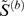 are the local within-class scatter matrix and the local between-class scatter matrix, respectively. 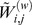 and 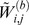 can be defined as



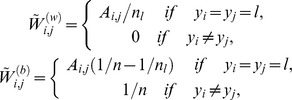
where *A_i,j_* is an affinity matrix, *A_i,j_* is large if *x_i_* and *x_j_* are ‘close’ and *A_i,j_* is small if *x_i_* and *x_j_* are ‘far apart’, *y_i_* is the label of *x_i_*, *n_l_* is the sample number of class *l*, and *n* is the total sample number.

A projection matrix ***L***_LFDA_ was first obtained using the LFDA method. The matrix ***L***_LFDA_ was then fed to the proposed REML method as the initial solution ***L***_0_ for further optimization. The complete REML procedure is listed in Table 3.

**Table 3:** Algorithm 2.

**Input:**
Testing query set 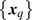 and their associated retrieved ranking list 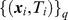 ; Parameter 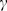 ; Maximum iteration number *t_max_*.
**Output:**
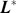 (Final solution of REML).
Initialize the projection 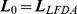 .
Estimate the step size of gradient: 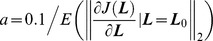 .
** For** *t* = 1,…, *t_max_*
▪ Calculate a stochastic gradient in Eq. (11) on a randomly sampled query with respect to 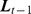
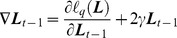 .
▪ Update 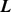 :
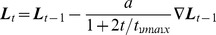 .

## Experimental Results

### 3.1 Retrieval Evaluation Measures

The retrieval evaluation measures for the proposed CBIR system are presented in this section. For a given number of retrieved images, the Precision and Recall are defined as:
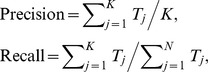
where 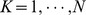 is the number of retrieved images and *N* is the total image number in the dataset. The precision-recall pairs for varying numbers of retrieved images are usually plotted in a precision-recall curve to evaluate the retrieval system. In the ranking list, precision at the top *n* retrieved images (Prec@n in short) is denoted by:



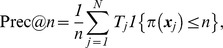
where 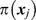 represents the position or rank of the retrieved image ***x*** *_j_* in the ranking list. The average precision (AP) is the average of the precisions at the positions where a relevant image exists:



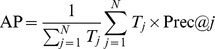



Finally, mAP is the mean AP over all of the queries:
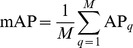
where *M* is the number of queries.

mAP and Prec@10 were used as the major quantitative measures to evaluate the performance of the algorithms. Furthermore, precision-recall curves were drawn for some cases to enable intuitive comparison.

### 3.2 Experimental Settings

In the experiments, the performance of the category retrievals of the brain tumors was evaluated through five-fold cross-validation method. The 233 patients were randomly partitioned into five subsets. Partitioning ensured that the slices from one patient do not exist in the training and test dataset at the same time. All experiments were repeated five times, and the final results were reported as the mean and standard deviation of the results from the individual runs. In each run, four subsets were used as a training dataset, and the remaining one was used as a test dataset. For the given training and test datasets, each image in the test dataset was adopted as a query to retrieve the training dataset to report the performance.

The parameter setting of all the algorithms tested in this study was carefully considered to obtain optimum performance during the experiments. Table 4 provides a summary of the parameter settings in the experiments.

**Table 4:** Parameter settings in the experiments.

Parameter	Description	Setting
*w*	Patch size (*w*×*w*)	7
*V*	Dictionary size	1000
*k*	Number of partition	9
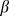	Parameter defined in Eq. (1)	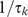 ( 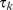 : maximal area among the *k* regions)
*t_max_*	Maximum iteration number defined in Algorithms 1 and 2	1000
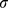	Scaling parameter defined in Eq. (7)	1
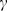	Regularization weight defined in Eq. (10)	0.0001
*d*	Dimensionality of transformed feature space of REML, MPP, and LFDA	3
*d*_CFML_	Dimensionality of transformed feature space of CFML	2
-	Coding method described in Section 2.1	soft-assignment
*p*	*p*-norm described in Section 2.1	∞

### 3.3 Experiments

#### 3.4.1 Parameter Optimization

**Figure 6:**
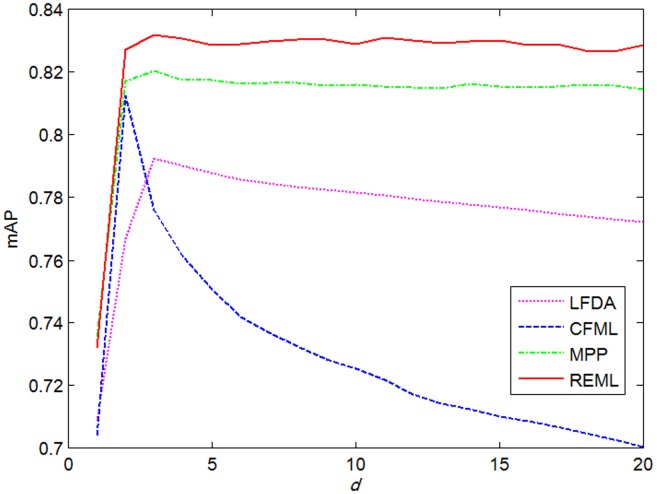
mAPs of different *d* values in LFDA, CFML, MPP, and REML.

The reduced dimensionality *d* of the transformed feature space is an important parameter for ***L***. We investigated the retrieval performance at different *d* values of LFDA, CFML, MPP, and the proposed REML to determine the optimal *d*. In addition, parameters *V*, *w*, and *k* were set to 1000, 7, and 0, respectively. The highest mAP values were achieved when *d* was set to 3 for LFDA, MPP, and REML, as shown in Figure 6. At the same time, the best performance of CFML was obtained when *d* was fixed to 2. Furthermore, REML and MPP were more robust than LFDA and CFML when *d* varied. The mAP values of REML and MPP were maintained at an approximately constant level, whereas the mAP values of LFDA and CFML significantly decreased when *d* was greater than 3 and 2, respectively. Therefore, the *d* values of LFDA, CFML, MPP, and REML were set to 3, 2, 3, and 3, respectively, in the subsequent experiments.

**Figure 7:**
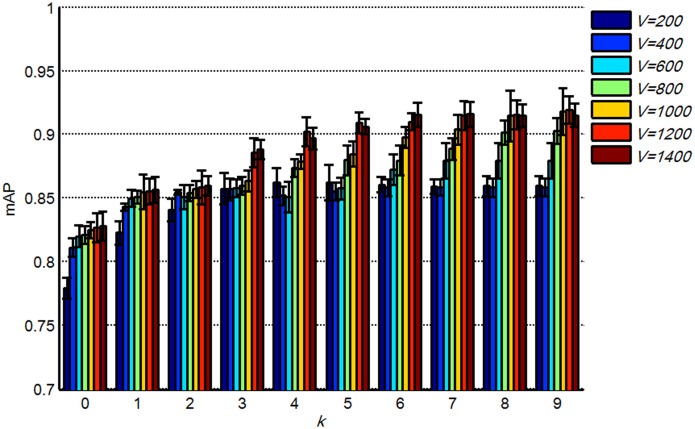
mAPs of the proposed method with different *V* and *k*; the bars show the means and standard deviations of mAPs.

The dictionary size *V* is a crucial parameter in the partition learning method. We investigated the retrieval performance at different *V* with the distance metric learned by REML to determine the optimal *V*. Parameters *d* and *w* were set to 3 and 7, respectively. In addition, *k* ranged from 0 to 9. Figure 7 shows that the retrieval performance was improved by increasing *V* with different *k* values. Large dictionaries contain numerous discriminative data, resulting in a higher retrieval performance compared with small dictionaries. However, in addition to high memory and computational costs, retrieval accuracy increased slowly at *V*>1000 (paired *t*-test *p* = 0.0095 when *V* ranged between 800 and 1000; paired *t*-test *p* = 0.049 when *V* ranged between 1000 and 1200). Therefore, to balance the trade-off between memory and computation costs and accuracy, the dictionary size *V* was fixed to 1000 in subsequent experiments.

**Figure 8:**
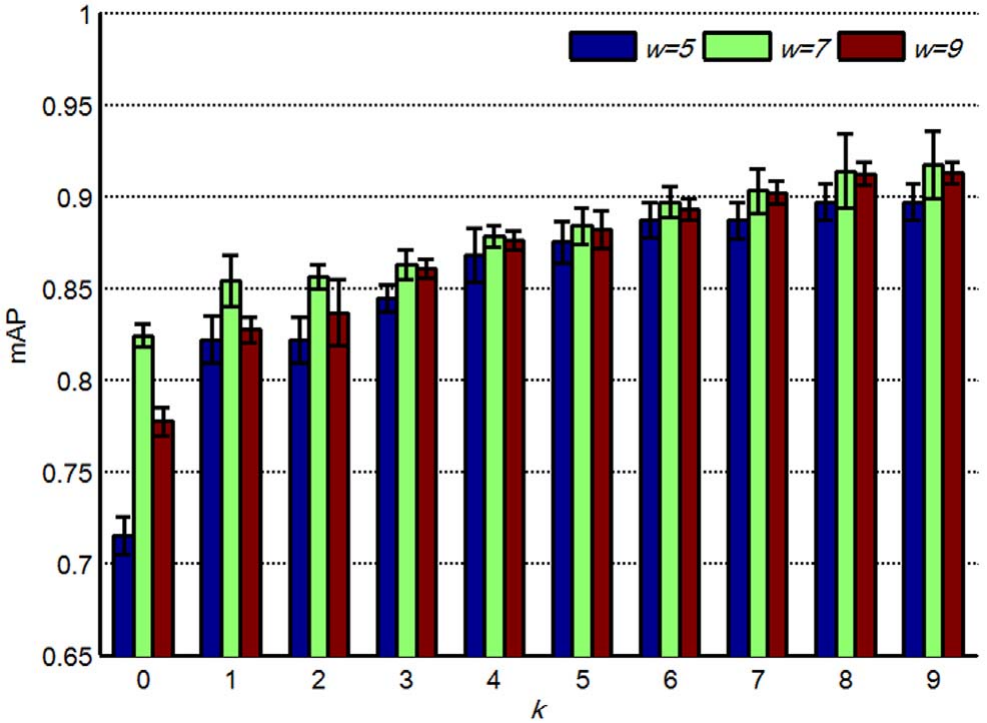
mAPs of the proposed method with different *w* and *k*; the bars show the means and standard deviations of mAPs.

The patch size *w* is another important parameter in the partition learning method. Three different patch sizes, 5, 7, and 9, with the distance metric learned by REML were used to assess the effect of patch size on the retrieval performance. Parameters *d* and *V* were set to 3 and 1000, respectively. In addition, *k* ranged from 0 to 9. Figure 8 shows that retrieval performance was improved by increasing *w* from 5 to 7 with different *k* values. In general, larger patches lead to more discriminative information for identifying different objects. However, in high-dimensional feature spaces associated with larger patches, a larger dictionary is necessary to construct the basis of the space, and more patches are required to train the model. As shown in Figure 8, the retrieval accuracy of *w* = 7 is higher than those of *w* = 5 (paired *t*-test *p* = 0.0068) and *w* = 9 (paired *t*-test *p* = 0.0081). Therefore, a moderate-sized patch is more applicable in the proposed method than patches of other sizes. Thus, *w* was set to 7 in subsequent experiments.

**Figure 9:**
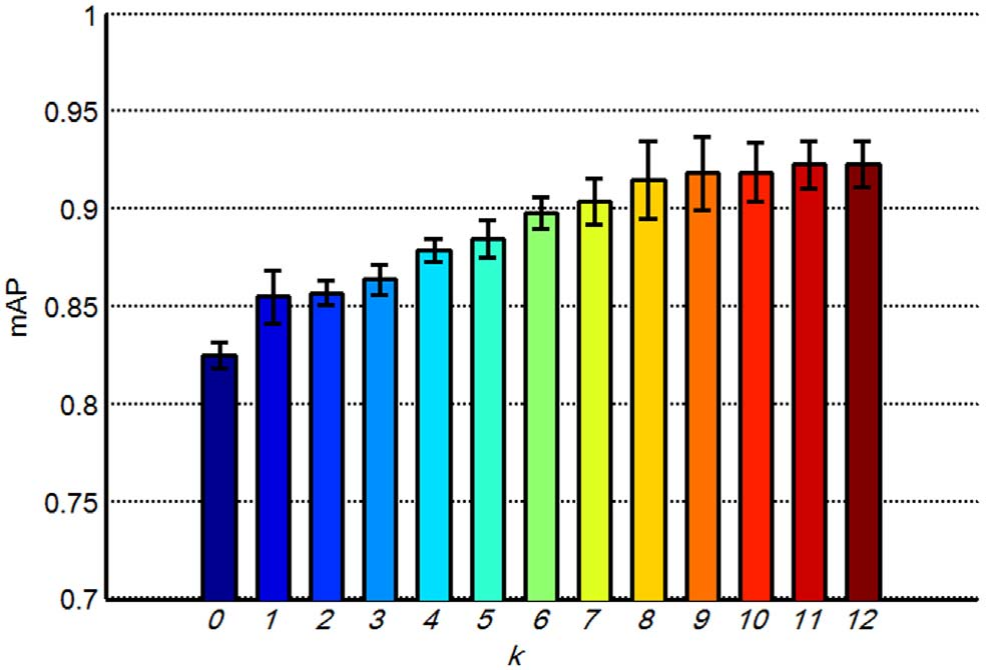
mAPs of the proposed method with different *k*; the bars show the means and standard deviations of mAPs.

**Figure 10:**
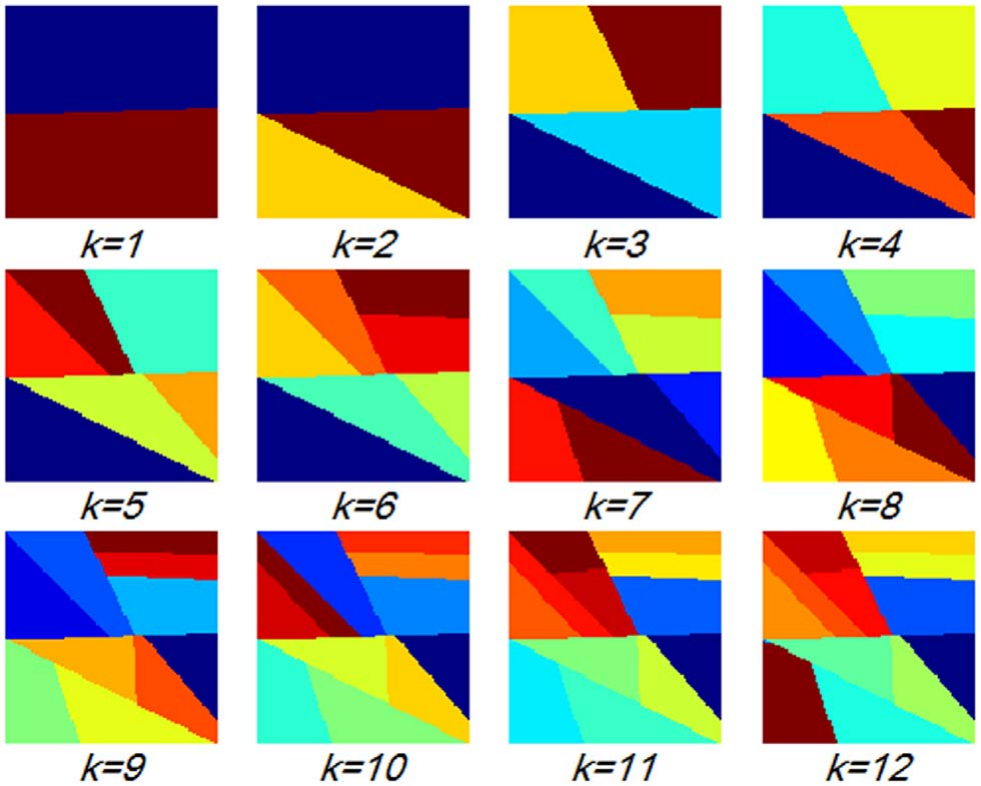
Partition patterns with different *k* when *V* = 1000 and *w = *7.

The number of partition *k* is the third parameter in the partition learning method that should be determined carefully. We varied *k* from 0 to 12 to investigate the variation of the retrieval performance with different *k* values by using the distance metric learned by REML. In addition, parameters *V*, *w*, and *d* were set to 1000, 7, and 3, respectively. Figure 9 shows the mAP values of different *k*. Increasing *k* was proven to be useful up to 9, as shown in Figure 9. Moreover, increasing *k* beyond 9 increased computational costs with no obvious improvement in the retrieval performance. Figure 10 shows the partition patterns with different *k* values. The larger *k* leads to finer partitions and obtains more discriminative information of the objects. However, when the images are too finely partitioned, the individual bins of the histograms may yield too few matches. This result may be why increasing *k* beyond 9 does not result in evident improvement in the retrieval performance. Figure 10 also shows that the spatial partitions of the brain tumor images are irregular, which may be attributed to the complex appearance of the brain tumors in the T1-weghted CE-MR Images (Figure 2).

#### 3.3.2 Evaluation of the Effectiveness of REML

**Figure 11:**
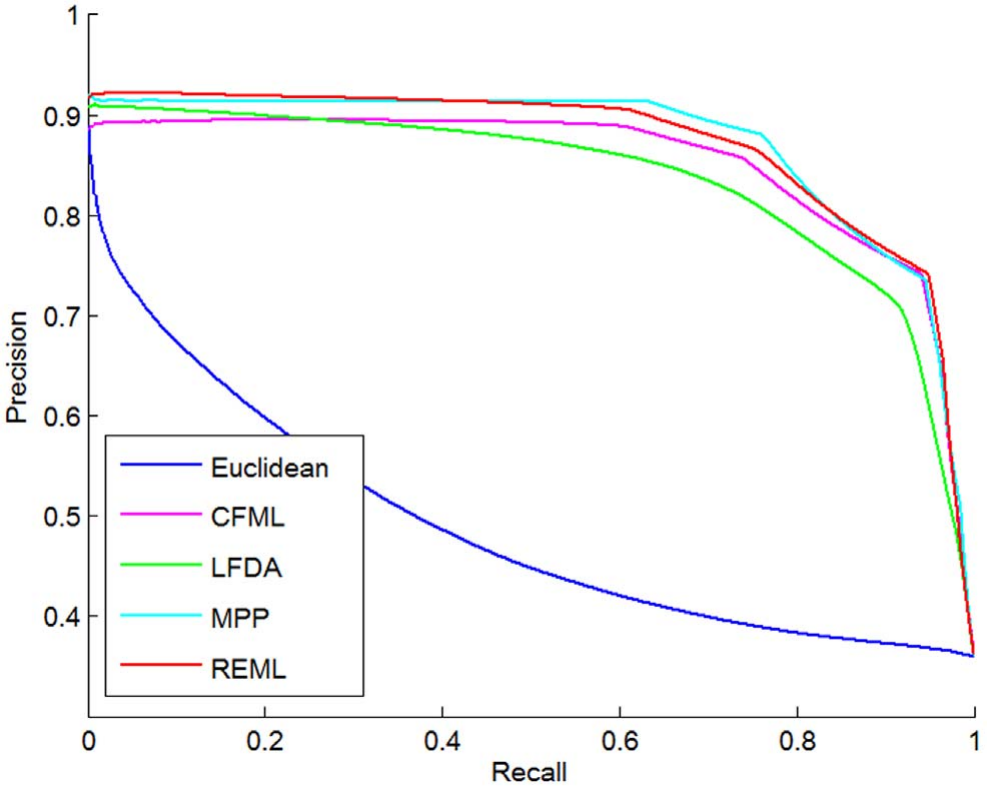
Precision-recall curves using different distance metric learning methods.

The BoVW histogram, which was constructed using the optimal parameters described above, was used as the feature input to learn different distance metrics. The retrieval performance of different distance metrics is summarized in Table 5 and Figure 11. As listed in Table 5, the proposed REML outperforms the Euclidean distance, CFML, LFDA, and MPP methods in terms of both mAP and Prec@10. Compared with the MPP, which had the second best performance, REML resulted in significant improvement (*p* = 0.03, paired *t*-test) in mAP (1.7% improvement from 90.1% to 91.8%). Compared with the Euclidean distance, CFML, LFDA, and MPP methods, the precision-recall curves in Figure 11 reveal that REML is the most effective distance metric learning method for improving the performance of the proposed CBIR system.

**Table 5:** Summary of the retrieval performance of different distance metrics (mean ± std %).

	Euclidean	CFML	LFDA	MPP	REML
mAP	46.5±0.1	86.6±1.0	89.2±1.9	90.1±2.47	91.8±1.8
Prec@10	79.4±0.2	89.3±0.2	90.9±0.2	91.7±0.1	93.1±0.1

#### 3.3.3 Evaluation of the Effectiveness of Partition learning

**Figure 12:**
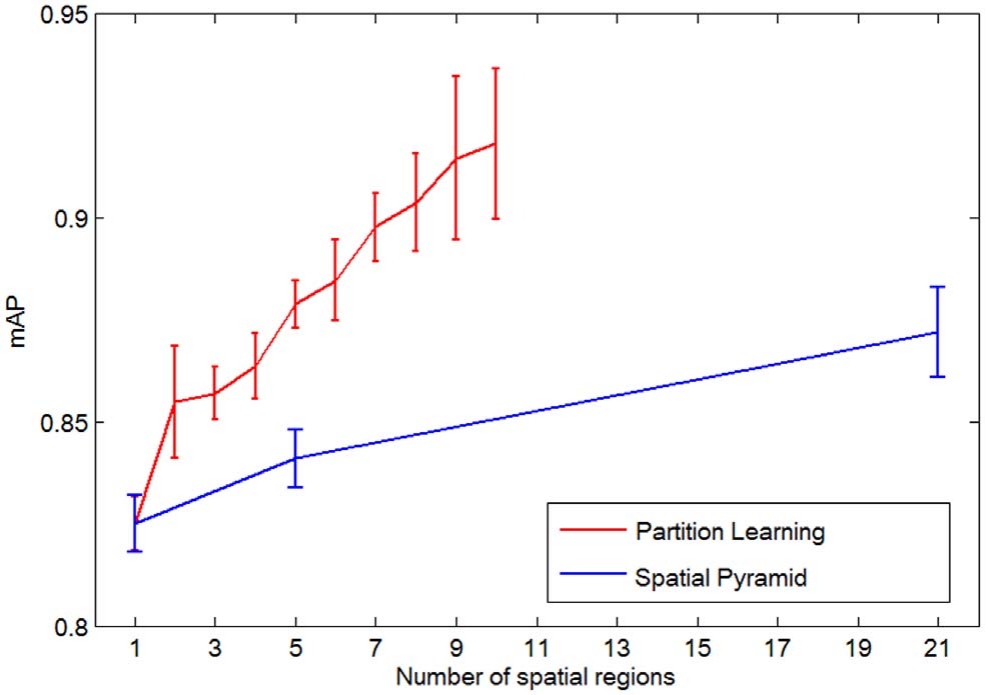
mAPs of the partition learning and spatial pyramid methods with different numbers of spatial regions.

The partition learning method was compared with the spatial pyramid method introduced in a previous study [25] to validate the performance of the proposed method. To provide a fair comparison, we fixed *V* = 1000, *w = *7, and *d* = 3, and used the distance matric learned by REML on both the partition learning method and the spatial pyramid method. In addition, *k* was set to 9 in the partition learning method, whereas the spatial level was set to 3 in the spatial pyramid method. Therefore, the number of spatial regions for pooling was 10 for the partition learning method and 21 for the spatial pyramid method (1+4+16 = 21), respectively. Furthermore, the partitions were performed on the rectangle ROIs for both the partition learning method and the spatial pyramid method. The comparison results are illustrated in Figure 12. In this experiment, the spatial pyramid method achieved a mean mAP value of 0.825±0.007 at pyramid level 0 (bounding box i.e., 1×1), 0.841±0.007 at pyramid levels 0 and 1 (1×1 and 2×2), and 0.872±0.011 at levels 0, 1, and 2 (1×1, 2×2 and 4×4). The performance decreased for spatial pyramid method when we go higher than level 2. As shown in Figure 12, the performance of the partition learning method with spatial regions equal to 10 (*k* = 9) outperformed the best performance (spatial regions = 21) of the spatial pyramid method. This result supports our claim that quantizing the image space by symmetric divisions is inappropriate to describe the spatial layout information and can be improved by the partition learning method. In addition, the computation and memory costs of the proposed method are much lower than those of the spatial pyramid method (feature dimensionality is 1000×9 = 9000 in the proposed method vs. 1000×21 = 21000 in the spatial pyramid method). These experimental results indicate that the partition learning method is more flexible than the spatial pyramid method and can better capture the discriminative information of the brain tumors in T1-weighted CE-MR Images.

#### 3.3.4 Retrieval Performance of the Proposed Method

The optimal parameters described above were used to construct the BoVW histogram and learn the distance metric for the proposed method. The retrieval performance of the proposed method for the different categories of brain tumors was evaluated, and the results are listed in Table 6. The retrieval performance of gliomas is higher than that of meningiomas and pituitary tumors, which supports the fact that the extracted features contain adequate discriminative information to distinguish gliomas from other tumor types (*p*<0.01, two sample *t*-test). The inferior performance of meningiomas may be due to the unbalanced distribution of samples in the dataset and their extremely similar appearance to pituitary tumors.

**Table 6:** Retrieval performance of the proposed method for different categories of brain tumor (mean ± std%).

Tumor category	mAP	Prec@10	Prec@20
Meningioma	81.7±2.7	88.9±2.9	87.9±2.9
Glioma	97.4±1.0	95.7±2.0	95.9±1.9
Pituitary tumor	93.2±1.7	94.5±2.1	94.2±2.0

#### 3.3.5 Comparison with Relevant Methods

To demonstrate the performance of the proposed method, it was compared with the performance of two other methods for brain tumor retrieval in T1-weighted CE-MR Images. Yang et al. [6] proposed a feature extraction method by capturing tumor margin information and designed a distance metric learning method by maximizing the mAP to retrieve brain tumors in the T1-weghted CE-MR Images. Huang et al. [7] used a fixed separation method combined with the BoVW model to capture the information of the intensity values in the tumor region and the intensity variation of the tumor-surrounding region for brain tumor retrieval. The dataset used in these two methods was identical to that used in the proposed method. For the proposed method, the optimal parameters described above were used to construct the BoVW histogram and learn the distance metric. Table 7 shows the comparison results of three methods. The retrieval results of the two compared methods are reported in the corresponding papers. The best result in each evaluation measure is shown in boldface in Table 7. The proposed method yielded the highest mAP and Prec@10 among the three methods, as listed in Table 7. This result demonstrates that the proposed method is effective and robust in retrieving brain tumor in T1-weighted CE-MR Images.

**Table 7:** Comparison of the proposed method with two relevant methods in terms of mAP and Prec@10 (%).

Methods	Yang et al. [6]	Huang et al. [7]	Proposed
mAP	87.3	91.0	**91.8**
Prec@10	89.3	91.7	**93.1**

#### 3.3.6 Computation Cost

A PC with Intel P4 3.0 GHz processor and 16 GB RAM was used as the workstation in this study. In the offline feature extraction step, learning an optimal partition with *V* = 1000, *w = *7, and *k* = 9 from a training dataset with 2450 images consumed 1 h. Furthermore, learning an optimized similarity metric using the extracted features and the proposed REML method consumed 2 min. In the online image retrieval step, the processing time was approximately 5 s.

## Discussion and Conclusion

Effective image features are crucial to produce satisfactory retrieval results. A partition learning algorithm is proposed to be integrated into the basic BoVW framework to extract discriminative features in this study. The coordinate descent-like iterations and greedy forward selection methods were used to optimize the objective function of the partition learning method. Therefore, the final solution of the partition learning method may vary for different initial conditions. In addition, the solution may offer a local optimal solution instead of a global optimal solution, which is a limitation in the partition learning method. However, the partition learning method still performed well during the experiments and achieved higher mAP values than other relevant methods (Table 7).

The major limitation of REML is that the learned distance metric is globally linear. In future work, we aim to learn multiple local projections for different regions in the feature space, similar to the multi-metric LMNN [43]. Moreover, the objective function of REML is optimized by the stochastic gradient descent algorithm; thus, the final solutions of ***L*** vary for different initial solutions and different query pairs randomly selected. An optional solution is to stabilize the REML solution by aggregation. Another competitive solution is to use the virtual convex cost function defined by the LambdaRank [48] algorithm and directly optimize the cost function with respect to the matrix ***M***, where 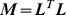 and ***M*** is a positive semidefinite matrix.

Although the proposed CBIR system is based on 2D CE-MR Images, the proposed system can be extended to 3D cases. As introduced in Section 2.2, 3D MR Images are difficult to obtain in the routine clinics of hospitals in China. Before extending the proposed method to 3D cases, the normalization of space resolutions in different directions among different subjects must be solved. Furthermore, 3D patches can be extracted to construct BoVW histograms and represent 3D regions. Region partitioning in 3D space is a more difficult task than region partitioning in 2D space because of the increasing complexity of the former. Instead of calculating the parameters in a line, parameters in a plane must be solved for region partition in 3D space.

Another aspect of this work that can be improved is the image intensity standardization step. The proposed method uses the N3 algorithm and min–max method to remove bias field artifacts and normalize image intensity, respectively. However, retrieval can benefit from sophisticated intensity standardization steps, such as inhomogeneity correction, noise removal filtering, and intensity standardization [49], [50]. Better intensity matching corresponds to more discriminative features.

The development of the CBIR system for brain tumors in MR Images has been widely investigated. Thus, placing our results in context helps to reveal the significance of our results. However, direct comparisons of quantitative retrieval results across publications are difficult and not always fair because of inconsistent tumor category and imaging protocol. Therefore, the proposed method was only compared with two brain tumor retrieval methods [6], [7], which were all performed using the same dataset. As listed in Table 7, mAP and Prec@10 of the proposed method are higher than those of Yang et al. [6] and Huang et al. [7].

In conclusion, this study presented a CBIR system for 2D brain tumor T1-weighted CE-MR Images. A partition learning method is integrated into the basic BoVW framework to extract discriminative features, which is very useful to the category retrieval task of brain T1-weighted CE-MR Images. A distance metric learning method is also proposed to reduce the sematic gap in the proposed CBIR system. The effectiveness of the partition learning and REML methods is demonstrated on a large T1-weighted CE-MR Image dataset of brain tumors.
